# Design and Application of a Mobile Ultra-Audio Frequency Electromagnetic Measurement System

**DOI:** 10.3390/s26072095

**Published:** 2026-03-27

**Authors:** Hongyu Ruan, Zucan Lin, Keyu Zhou, Yongqing Wang, Qisheng Zhang, Hui Zhang

**Affiliations:** 1 School of Geophysics and Information Technology, China University of Geosciences, Beijing 100083, China; 2 Institute of Acoustics, Chinese Academy of Sciences, Beijing 100083, China

**Keywords:** CSRMT, signals of opportunity, electromagnetic receiver, ultra-audio frequency, mobile design

## Abstract

Although high-frequency electromagnetic methods, such as Radio Magnetotellurics (RMT) and Controlled-Source Radio Magnetotellurics (CSRMT), are highly effective for shallow-to-medium depth exploration, deploying traditional transmitter–receiver setups remains labor-intensive and significantly slows down large-scale surveys. To overcome these logistical bottlenecks, we developed a mobile Ultra-Audio Frequency Electromagnetic (UAEM) measurement system. While the hardware is designed with dual-mode capabilities supporting conventional controlled-source operations, this paper specifically focuses on its application in a Signals of Opportunity (SOOP) mode. By utilizing pre-existing, stable anthropogenic signals, including Amplitude Modulation (AM) broadcasts and naval very low frequency communications, the system effectively functions as a broadband RMT receiver. Technical evaluations demonstrate that the instrument operates across a 1 Hz to 1000 kHz bandwidth with a high sampling rate of 2.5 MHz. Furthermore, it achieves a dynamic range of 143 dB and maintains an apparent resistivity measurement accuracy of better than 3%. Thanks to its modular, vehicle-towed design, the UAEM system enables continuous, on-the-move data acquisition wherever ambient field sources are available. This approach eliminates the need for dedicated transmitter deployment, fundamentally reducing exploration costs and boosting overall survey efficiency.

## 1. Introduction

In terrestrial exploration, electrical and electromagnetic (EM) methods are commonly employed to investigate geological structures, typically by measuring electrical conductivity to infer properties such as porosity and lithology [1]. The magnetotelluric (MT) method, an electromagnetic exploration technique that utilizes natural-source fields, was initially proposed by Tikhonov [2] and subsequently adapted for electrical exploration by Cagniard in 1953 [3]. Operating over an extremely broad frequency band, it finds widespread application in mineral and hydrocarbon exploration, as well as in regional tectonic studies [4,5]. However, because the natural-source electromagnetic fields are weak in amplitude and random in polarization, the MT method is susceptible to noise interference [6]. Consequently, the Controlled-Source Audio-frequency Magnetotelluric (CSAMT) method was developed. This technique employs a grounded square-wave transmitter to induce a transient electromagnetic field in the earth, while receivers synchronously measure the frequency-dependent primary and secondary fields to perform electromagnetic sounding [7]. The Controlled-Source Radio Magnetotelluric (CSRMT) method builds upon traditional MT principles by utilizing an artificial transmitter to generate a primary field that is more stable and powerful than that of natural sources, thereby generally improving survey precision. By analyzing the spatial distribution of the transmitted signal, this geophysical technique compensates for the energy deficiency of natural fields in the high-frequency band (10 kHz–1 MHz) and is primarily applied to near-surface investigations in hydrogeology and environmental engineering [8]. As a major branch of geophysical survey methods, artificial-source electromagnetic techniques have achieved significant advancements over the past decade. These methods are systematically categorized into four modes: airborne (AEM), ground-to-air (GAEM), ground-based (GEM), and marine (MEM) [9]. Signals of Opportunity (SOOPs) are defined as signals not originally intended for navigation or electromagnetic exploration purposes [10]. Although SOOPs can theoretically be used for positioning, navigation, and timing (PNT), conventional EM exploration methods have not leveraged them for geological surveying. Furthermore, traditional approaches typically require the dedicated deployment of a transmitter to generate an artificial source field based on survey requirements [11]. This practice not only increases the costs associated with equipment deployment and maintenance but also reduces survey efficiency. Moreover, the fixed-source configuration restricts the mobility of the equipment, making large-scale continuous surveys challenging.

Our research group has been dedicated to the development of electromagnetic (EM) measurement instrumentation based on the CSRMT method. We have successfully developed a suite of components, including a miniaturized low-frequency transmitter module, a high-frequency electric dipole source module, high-frequency induction-coil magnetic field sensors, SQUID magnetometers, and a prototype Ultra-Audio frequency EM (UAEM) receiver. All these components have reached the engineering prototype stage [12,13]. In our previous survey missions, the deployment of artificial sources was a mandatory step for geological investigation. Therefore, this paper introduces a mobile, dual-mode UAEM system. The system is compatible with dedicated transmitters for operation in a “controlled-source mode”. It can also utilize pre-existing Signals of Opportunity (SOOPs), such as amplitude modulation (AM) broadcast signals, Very Low-Frequency (VLF) naval communication transmissions, and national time service signals, for exploration. Within the transmission periods and coverage areas of these sources, the vehicle-mounted UAEM system can perform mobile surveys. Unlike conventional artificial-source EM methods, this system obviates the need for on-site transmitter deployment. This approach significantly enhances survey efficiency while concurrently reducing the costs associated with the setup and maintenance of transmitters. It provides a highly efficient and low-cost complementary or alternative solution under specific geological conditions, particularly for surveys in areas with stable ambient field coverage, shallow exploration targets, or where challenging ground conditions (e.g., urban paved surfaces) make electrode installation difficult. This SOOP-based exploration methodology effectively transforms ubiquitous communication signals into a valuable geophysical measurement tool.

The remainder of this paper is organized as follows. Section 2 introduces the fundamental principles and theoretical foundation of the mobile Ultra-Audio Frequency Electromagnetic (UAEM) measure system. Section 3 presents the detailed system design, encompassing the overall architecture, the analog and interface boards, the main control board, and the software architecture. In Section 4, the system’s performance is experimentally validated and analyzed through a series of laboratory performance tests and a field experiment, which includes a comparative analysis against a conventional instrument. Finally, Section 5 concludes the paper by summarizing the key findings and outlining future research directions.

## 2. Theory and Method

### 2.1. Principles of Mobile UAEM Electromagnetic Measurement System

**Figure 1:**
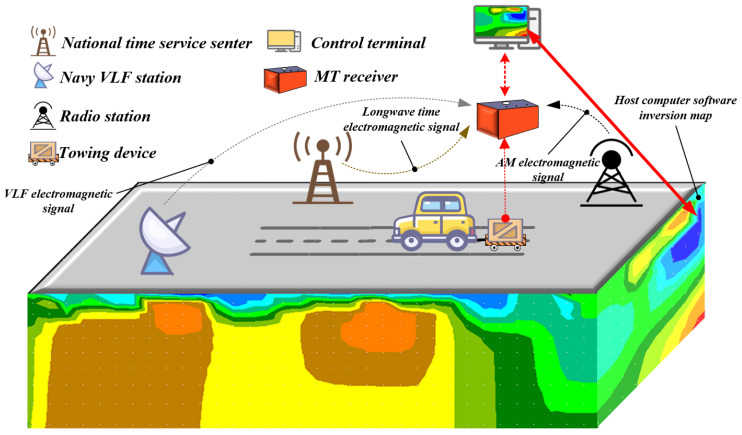
Schematic diagram of the UAEM measurement system.

Controlled-Source Radio Magnetotellurics (CSRMT) is a hybrid geophysical method derived from the integration of Radio Magnetotellurics (RMT) and Controlled-Source Magnetotellurics (CSMT). This ultra-audio frequency technique is primarily employed for near-surface investigations [14,15,16]. The method utilizes a deployable, artificial transmitter to generate an electromagnetic field, and resolves subsurface structures by measuring the resulting far-field components. Its operating frequency band in the ultra-audio range (1–1000 kHz) is significantly higher than that of conventional magnetotellurics, enabling an investigation depth that spans from several meters to hundreds of meters [16,17]. Given the technical challenges associated with generating controlled-source signals above 10 kHz, exploiting existing signals from Very Low-Frequency (VLF) transmitters and radio broadcasts has emerged as an advantageous alternative. For instance, Pedersen et al. experimentally demonstrated that stable magnetotelluric transfer functions could be calculated in Europe using far-field signals from radio transmitters in the 10–250 kHz frequency range [18]. Drawing upon these principles, the mobile Ultra-Audio Electromagnetic (UAEM) measurement system presented in this paper (Figure 1) operates by receiving signals from existing anthropogenic sources, such as AM broadcast stations, naval VLF communication signals, and national longwave time-code broadcasts. Furthermore, for mobile acquisition, the entire UAEM system is mounted on a platform towed by a vehicle. This platform is constructed from non-metallic materials, such as wood, to prevent electromagnetic interference with the system’s magnetic sensors.

### 2.2. Theoretical Basis for the System

The CSRMT method involves the measurement of [18] controlled-source electromagnetic fields within the ultra-audio frequency band (1–1000 kHz). By acquiring orthogonal, horizontal components of the electric (E) and magnetic (H) fields, the technique allows for the derivation of impedance amplitude (i.e., apparent resistivity) and phase, which are subsequently used for inversion [19]. According to frequency-domain electromagnetic sounding principles, higher-frequency measurements enhance vertical resolution [20]. The UAEM system presented herein, which is based on the CSRMT method, features an operational bandwidth extending from 1 Hz to 1000 kHz, a sampling rate of 2.5 MHz, and a dynamic range of 143 dB. The system is designed to utilize a variety of pre-existing anthropogenic field sources, including AM broadcast signals, VLF transmissions from naval stations, and timing signals from the National Time Service Center (NTSC). When operating in the far-field zone—defined as a distance from the transmitter exceeding approximately three skin depths—the electromagnetic field can be approximated as a plane wave. Under this plane-wave condition, the apparent resistivity is calculated from the orthogonal E and H components using Equation (1). This approach offers superior performance in far-field applications compared to conventional magnetotellurics (MT), primarily due to the higher signal-to-noise ratio provided by the artificial source [21]. By measuring E and H across a range of frequencies, a frequency-dependent apparent resistivity curve is obtained. This curve can then be inverted to resolve the subsurface electrical structure at various depths.

(1) $$\rho =\frac{1}{\mu \omega }\frac{{\left|E_{\varphi }\right|}^{2}}{{\left|H_{\gamma }\right|}^{2}}$$

where ρ denotes the apparent resistivity (in Ω·m), which provides a bulk measure of the electrical properties of the subsurface. Here, μ is the magnetic permeability of the medium (in H/m); for most geological materials it is commonly approximated by the permeability of free space, $\mu_{0}\approx 4\pi \times 10^{-7}\;H/m$. The quantity ω is the angular frequency (in rad/s), related to the conventional frequency *f* (in Hz) through ω=2πf. Moreover, $E_{\varphi }$ and $H_{\gamma }$ are the amplitudes (phasor magnitudes) of two orthogonal horizontal components of the electric and magnetic fields, respectively, with units of V/m and A/m. By measuring the ratio of these field components across a range of frequencies, a frequency-dependent apparent resistivity curve is constructed, which is subsequently inverted to resolve the subsurface electrical structure [22].

Furthermore, the theoretical limit of investigation depth for the broadband sensors is intrinsically governed by the electromagnetic skin depth. The skin depth δ is defined as the distance at which the amplitude of an electromagnetic plane wave attenuates to 1/e (approximately 37%) of its surface value, and is mathematically expressed as:

(2) $$\delta \approx 503\sqrt{\rho /f}$$

where δ is the theoretical depth of investigation (in m), ρ is the continuous apparent resistivity of the half-space (in Ω·m), and f is the carrier frequency (in Hz). Because the proposed UAEM system specifically targets high-frequency SOOP signals—ranging from the VLF band (10–30 kHz) up to the AM broadcast band (up to 1000 kHz)—the penetration depth is inherently restricted. In typical urban environments with conductive overburden, this theoretical limit confines the effective investigation strictly to the extremely shallow subsurface (typically 0–50 m). However, this physical limitation in penetration depth is precisely what endows the UAEM system with exceptionally high spatial resolution, making it uniquely advantageous for precise mapping of shallow urban engineering targets, such as underground pipelines and micro-voids.

## 3. Design Scheme of UAEM Measurement System

### 3.1. System Overview

**Figure 2:**
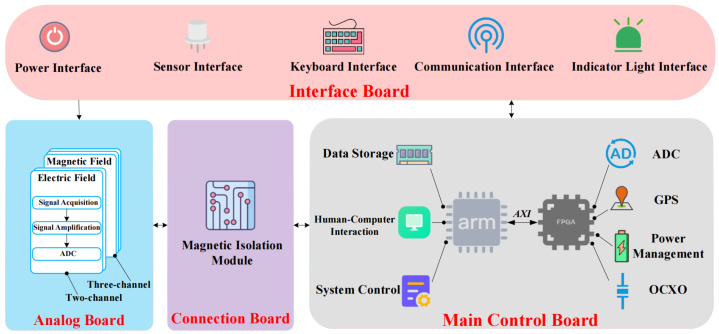
The overall hardware architecture diagram.

To achieve multi-channel data acquisition and transmission of electric and magnetic field components, while meeting the functional requirements of the receiver and current technological capabilities, the overall hardware architecture of the audio-frequency controlled-source electromagnetic receiving system was designed, as shown in Figure 2. The architecture consists of four main components: interface board, analog board, connection board, and main control board. Among these components, all circuit boards except the interface board are fixed within the mechanical enclosure of the receiver.

### 3.2. Analog Board Design

**Figure 3:**
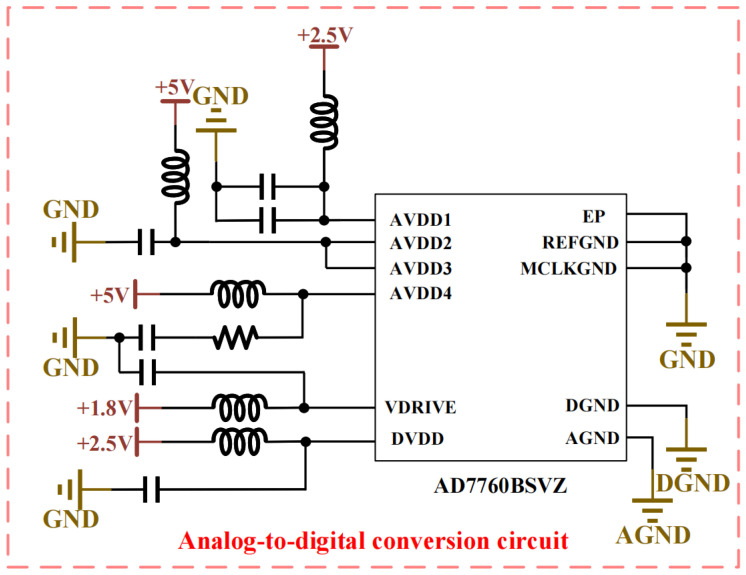
Schematic diagram of the Analog-to-digital conversion circuit.

The analog board incorporates analog-to-digital conversion functionality for multi-channel electric and magnetic field components, utilizing the AD7760 ADC (Analog Devices, Inc., Wilmington, MA, USA) and its peripheral circuits, as shown in Figure 3 [23]. To ensure power stability and low-noise characteristics, inductors and decoupling capacitors are implemented in the power supply and grounding sections of the AD7760, thereby enhancing ADC performance. Furthermore, the decoupling treatment of power and ground pins ensures isolation between analog and digital sections while maintaining a low-noise environment. The digital interface connects to the MPSoC (AMD, San Jose, CA, USA) on the main control board via the connection board. The AD7760 ADC provides 24-bit-resolution sampling data with a maximum sampling rate of 2.5 MSPS and achieves a signal-to-noise ratio of 100 dB at 2.5 MSPS, fully meeting the sampling requirements of the audio-frequency controlled-source electromagnetic receiving system at its maximum operating frequency of 1000 kHz.

**Figure 4:**
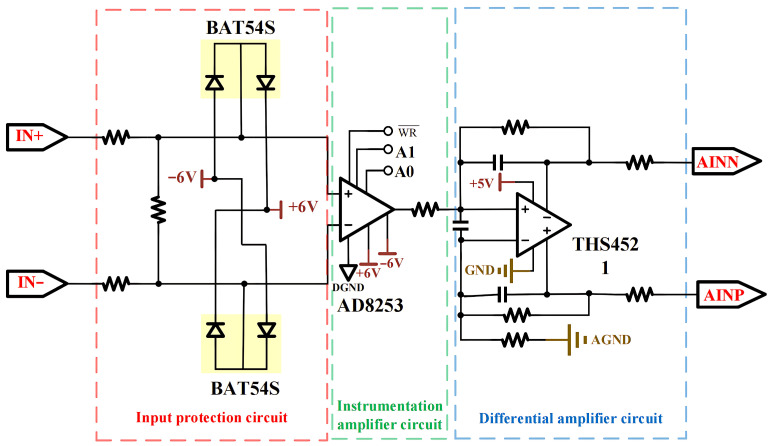
Schematic diagram of signal conditioning circuit.

The signal conditioning circuit on the analog board is shown in Figure 4. The input signal first passes through current-limiting resistors and Schottky diodes for input protection, effectively preventing overvoltage damage to subsequent circuits. The AD8253 (Analog Devices, Inc., Wilmington, MA, USA) is selected as the programmable gain amplifier (PGA), which is a high-precision, wide-bandwidth programmable gain instrumentation amplifier suitable for applications requiring high precision and bandwidth, such as data acquisition systems, medical equipment, and industrial control systems. With a programmable gain range of 1 to 1000 and a signal-to-noise ratio of up to 90 dB, it significantly enhances the circuit’s capability to capture weak signals. Various protection and filtering measures are implemented in the signal conditioning circuit to ensure signal integrity and circuit stability.

### 3.3. Interface Board Design

**Figure 5:**
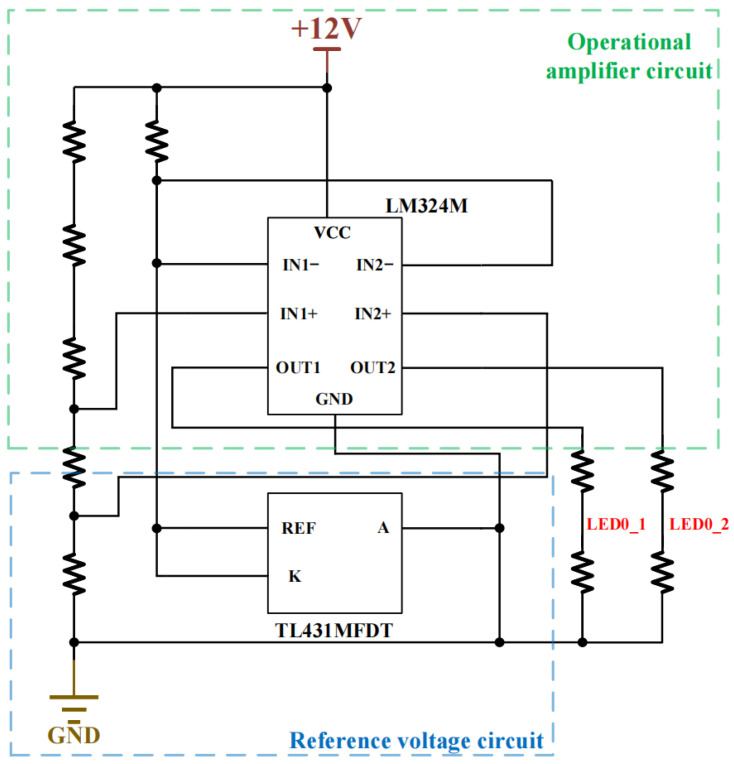
Schematic diagram of power quantity display circuit.

The interface board integrates power interfaces, sensor interfaces, communication interfaces, LED indicators, and keyboard interfaces. The power interface is responsible for connecting to the external 12 V power supply and distributing power to the entire system through the power distribution network. The sensor interface consists of magnetic field sensors and electrode terminals, which are connected to the analog channel input terminals on the analog board. The communication interface, LED indicators, and keyboard interface, primarily serving human–machine interaction functions, are connected to the Multi-Processor System-on-Chip (MPSoC) on the main control board through internal wiring. Additionally, this module features overcurrent protection and power monitoring capabilities, with part of the power display circuit structure shown in Figure 5.

### 3.4. Main Control Board Design

**Figure 6:**
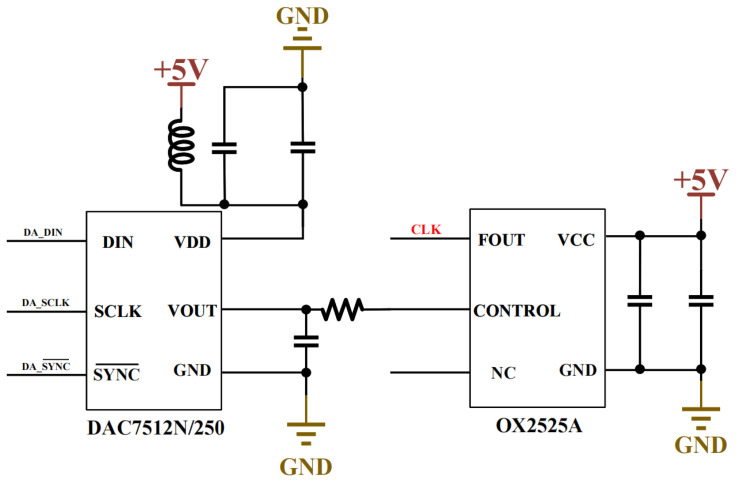
Schematic diagram of constant-temperature crystal calibration circuit.

The main control board employs the ZU3EG MPSoC (AMD, San Jose, CA, USA) as its core chip, which integrates both ARM and FPGA components with clearly defined roles in the system. The FPGA section primarily handles analog-to-digital conversion control, GPS data reception and parsing, OCXO calibration, and system power management. The ARM section is responsible for key tasks including system process control, human–machine interaction, and data storage. Additionally, the main board features circuits for storing program code on Micro-SD cards, GPS circuits for time synchronization, and an OCXO circuit providing stable clock signals. The solid-state drive, with read/write speeds up to 3.5 GB/s, fully meets the requirements for real-time multi-channel high-sampling-rate waveform storage (40 MB/s) during electromagnetic exploration. The OCXO circuit consists of a DAC circuit and a voltage-controlled OCXO, where the DAC generates precise voltage signals to calibrate the OCXO. Figure 6 shows part of the OCXO calibration circuit structure.

### 3.5. Software Architecture Design

The core of the electromagnetic survey system’s software and firmware architecture resides within the Field-Programmable Gate Array (FPGA) fabric of the Multiprocessor System-on-Chip (MPSoC), which communicates with the ARM processing system via the AXI bus. The FPGA’s primary responsibilities include controlling the analog-to-digital converters (ADCs), receiving and parsing the digitized data streams, processing GPS data, calibrating the Oven-Controlled Crystal Oscillator (OCXO), and executing system-level power management. The ARM processing system, in turn, manages the overall workflow of the acquisition station, handles human–machine interaction (HMI), and oversees data storage operations. The configuration of acquisition parameters and start/stop control is implemented by the ARM processor through read/write operations on dedicated registers within the FPGA. Following initial processing of the ADC data, the FPGA streams the resulting data to the ARM processor. The ARM subsequently stores the data onto an external Solid-State Drive (SSD) and simultaneously transmits it in real-time to a host computer via Ethernet. To suppress the broadband noise inherent in mobile surveys and enhance the signal-to-noise ratio (SNR), a high-efficiency, real-time digital signal processing (DSP) chain is implemented within the MPSoC’s FPGA fabric. This processing chain employs cascaded digital filtering prior to data decimation (downsampling), a strategy that effectively prevents spectral aliasing. This process removes high-frequency noise while ensuring the distortion-free extraction of the useful signal.

## 4. Experimental Verification and Analysis

### 4.1. Laboratory Performance Tests

**Figure 7:**
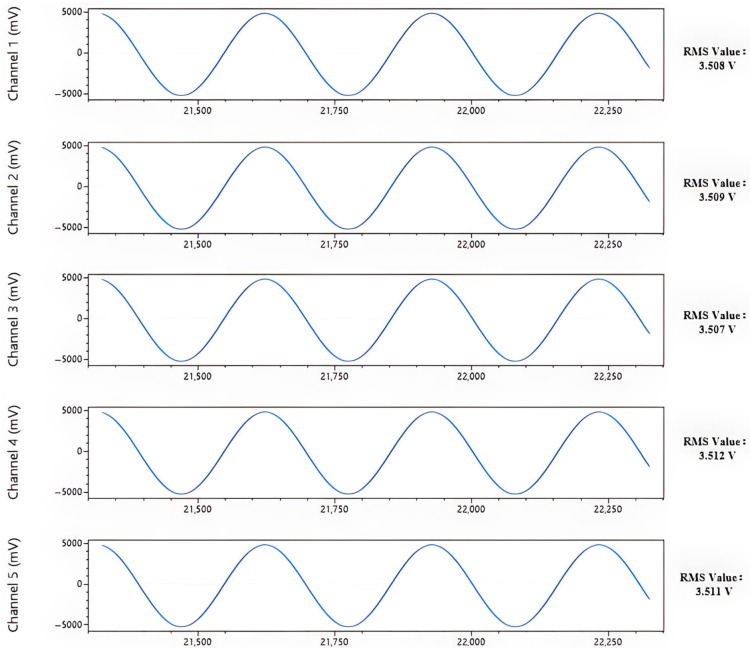
Maximum non-distorted output waveform.

**Figure 8:**
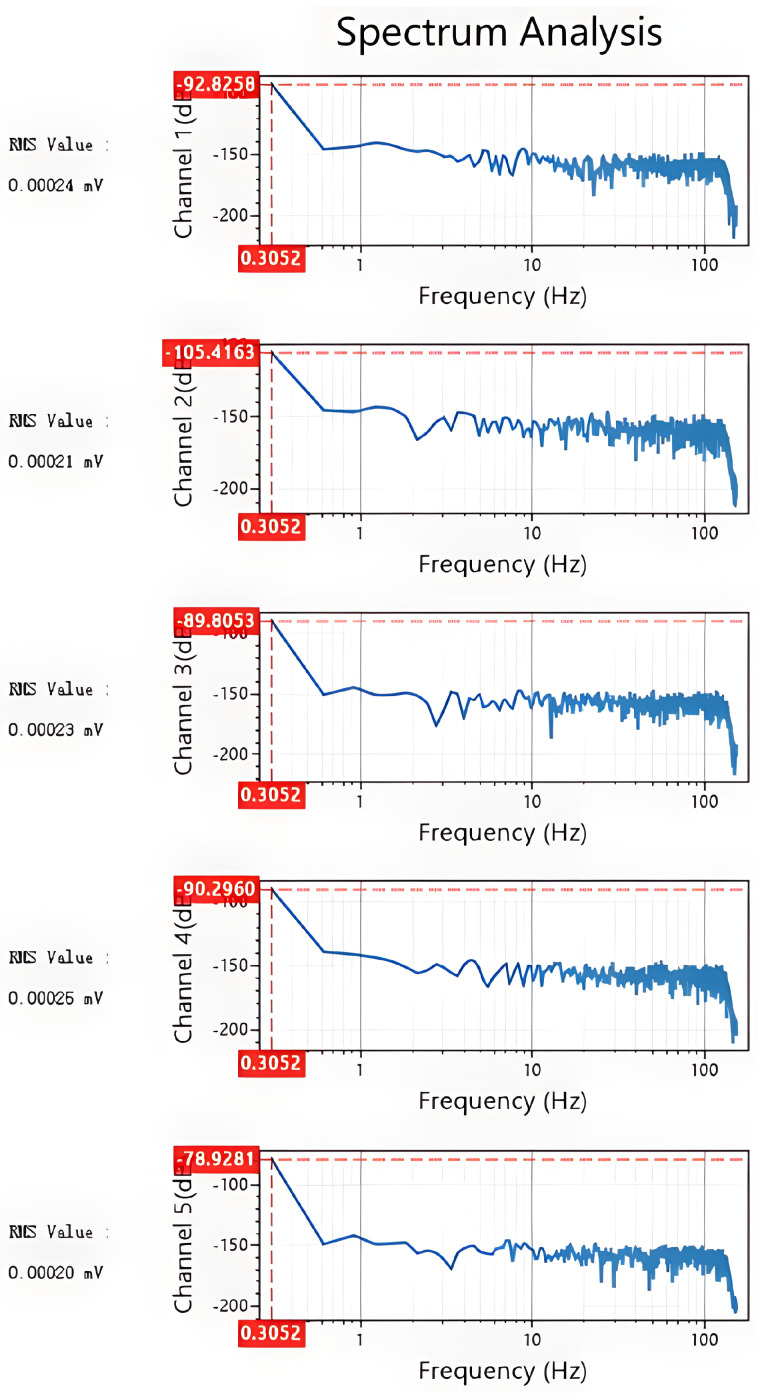
System noise floor with shorted input terminals.

To comprehensively and quantitatively evaluate the core performance of the developed system, a series of rigorous laboratory tests was conducted. First, a frequency response test was performed. A standard 1 V peak-to-peak sinusoidal signal was applied to the input across a swept frequency range. The maximum input signal achievable without significant clipping distortion was determined to be 3.5 Vrms, with its corresponding output waveform depicted in Figure 7. The results of the frequency sweep, summarized in Table 1, confirmed that the signal attenuation remained below 3 dB across the entire 1 Hz to 1000 kHz band, thus demonstrating the system’s excellent broadband response capabilities. Subsequently, the system’s dynamic range was evaluated. By shorting the input terminals to ground, the system’s noise floor was measured at 0.25 µVrms, as illustrated in Figure 8. Following the definition of dynamic range (DR = 20 log10(Vmax/Vnoise)), the measured dynamic range was calculated to be 143 dB. Collectively, these test results verify that the UAEM survey system successfully meets its design specifications for key performance metrics, including frequency response, noise suppression, and dynamic range. This establishes a robust hardware foundation for high-fidelity, wide-dynamic-range field data acquisition [13].

### 4.2. Field Survey Experiment

**Figure 9:**
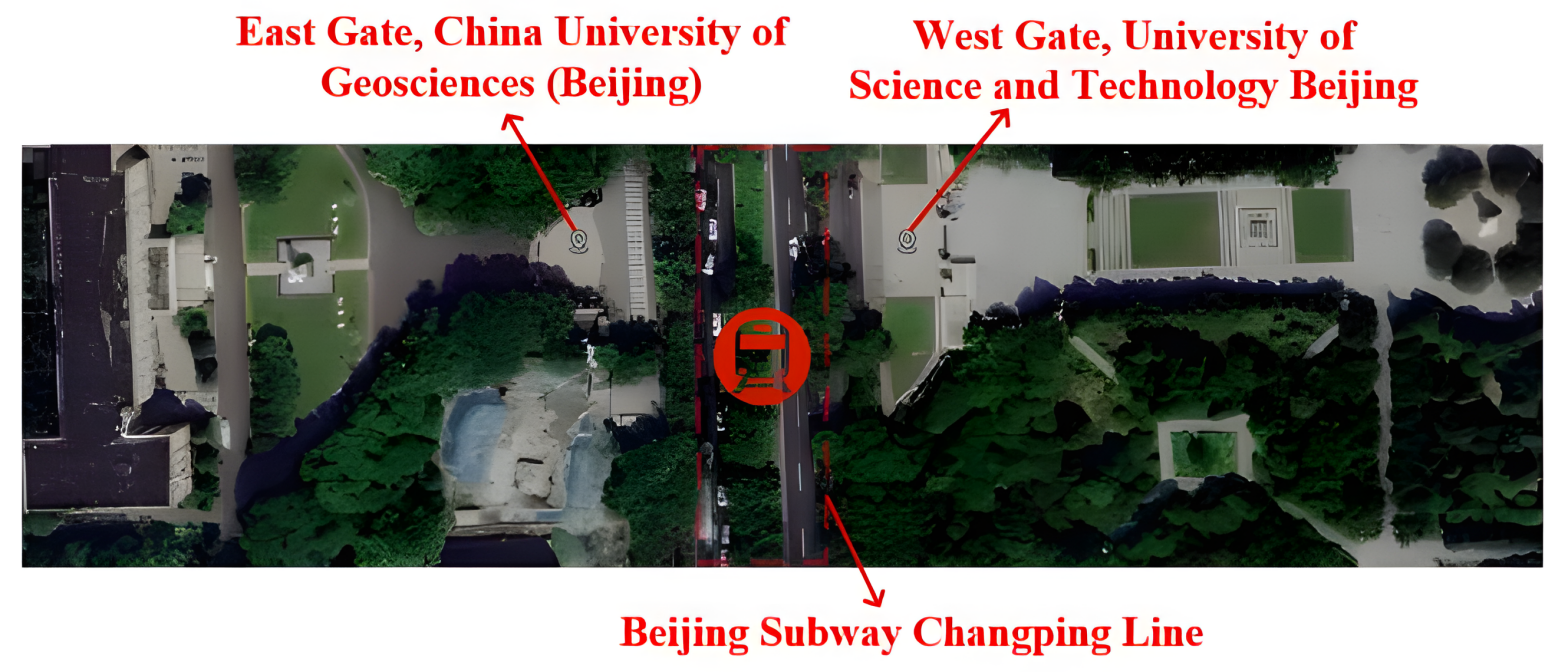
Location map of the Beijing Metro Changping Line tunnel.

To comprehensively validate the in-field performance of the UAEM measurement system, a field survey with a well-defined objective was designed and implemented. The survey area was situated along a profile between the East Gate of China University of Geosciences, Beijing (CUGB), and the West Gate of the University of Science and Technology Beijing (USTB) (see Figure 9). The primary objective of this experiment was to use electromagnetic methods to precisely locate and image the structure of the Beijing Metro Changping Line tunnel, which is buried at a depth of approximately 15–20 m [24,25]. Owing to its construction from reinforced concrete and a metallic lining, the tunnel presents a strong electrical contrast against the surrounding host soil. This characteristic makes it an ideal exploration target for validating the effectiveness of the developed system.

**Figure 10:**
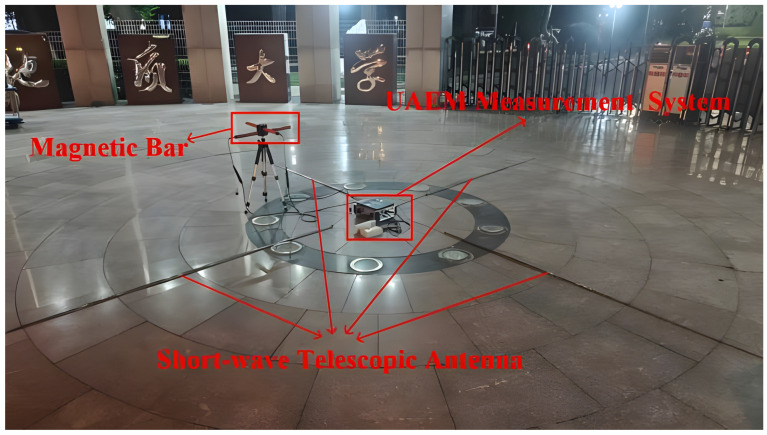
The UAEM measurement system in field operation.

The significance of this experiment lies in demonstrating that the adopted “Signals of Opportunity (SOOP) plus mobile survey” approach is particularly well-suited for rapid, non-destructive investigations in urban environments. This methodology obviates the need for deploying an artificial transmitter, thereby effectively circumventing the challenges of limited operational space and heavy traffic common to such settings. We established a survey line perpendicular to the axis of the metro tunnel, along which data were acquired at 14 measurement stations. During deployment, the UAEM system was configured with two induction-coil magnetometers and four shortwave whip antennas; the field setup is illustrated in Figure 10. The magnetometers employed were the in-house-developed UAEM induction-coil sensors. These sensors achieve an extremely low noise floor across the 1 Hz–1000 kHz ultra-wideband and provide a flat frequency response, which is accomplished through a core negative feedback technique. This ensures the high-fidelity, high-precision reception of faint electromagnetic signals originating from the subsurface target. For electric field measurements, 5.6-m vertically polarized whip antennas were used. This antenna type was selected for its favorable omnidirectional reception characteristics in the VLF-HF band and its ease of deployment.

**Figure 11:**
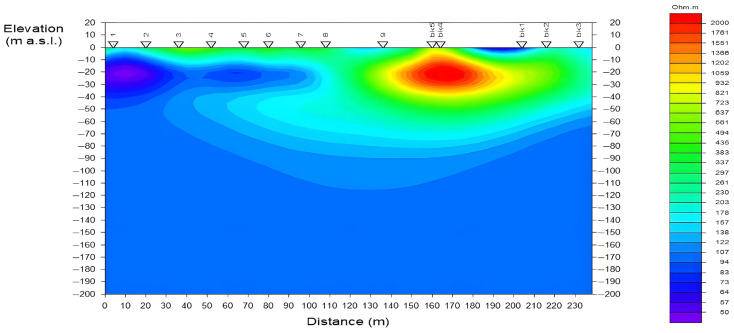
Inverted 2D resistivity model of the Changping survey line.

The multi-channel electromagnetic data acquired in the field were processed and subsequently subjected to two-dimensional (2D) inversion, yielding the subsurface resistivity profile presented in Figure 11. The profile clearly delineates a prominent high-resistivity anomaly (resistivity > 1000 Ω·m), indicated by the red and yellow contours, located at a horizontal distance of 150–200 m and a depth of 10–40 m. The position, geometry, and burial depth of this anomaly are in excellent agreement with the known location of the Changping Line metro tunnel. This resistive feature is a direct geophysical expression of the strong electrical contrast between the tunnel’s air-filled void and its dry lining, and the surrounding water-saturated, low-resistivity host soils (represented by the blue-green background). This successful outcome provides a compelling validation of the system’s effectiveness in using signals of opportunity for the high-precision detection of subsurface targets in urban settings.

**Figure 12:**
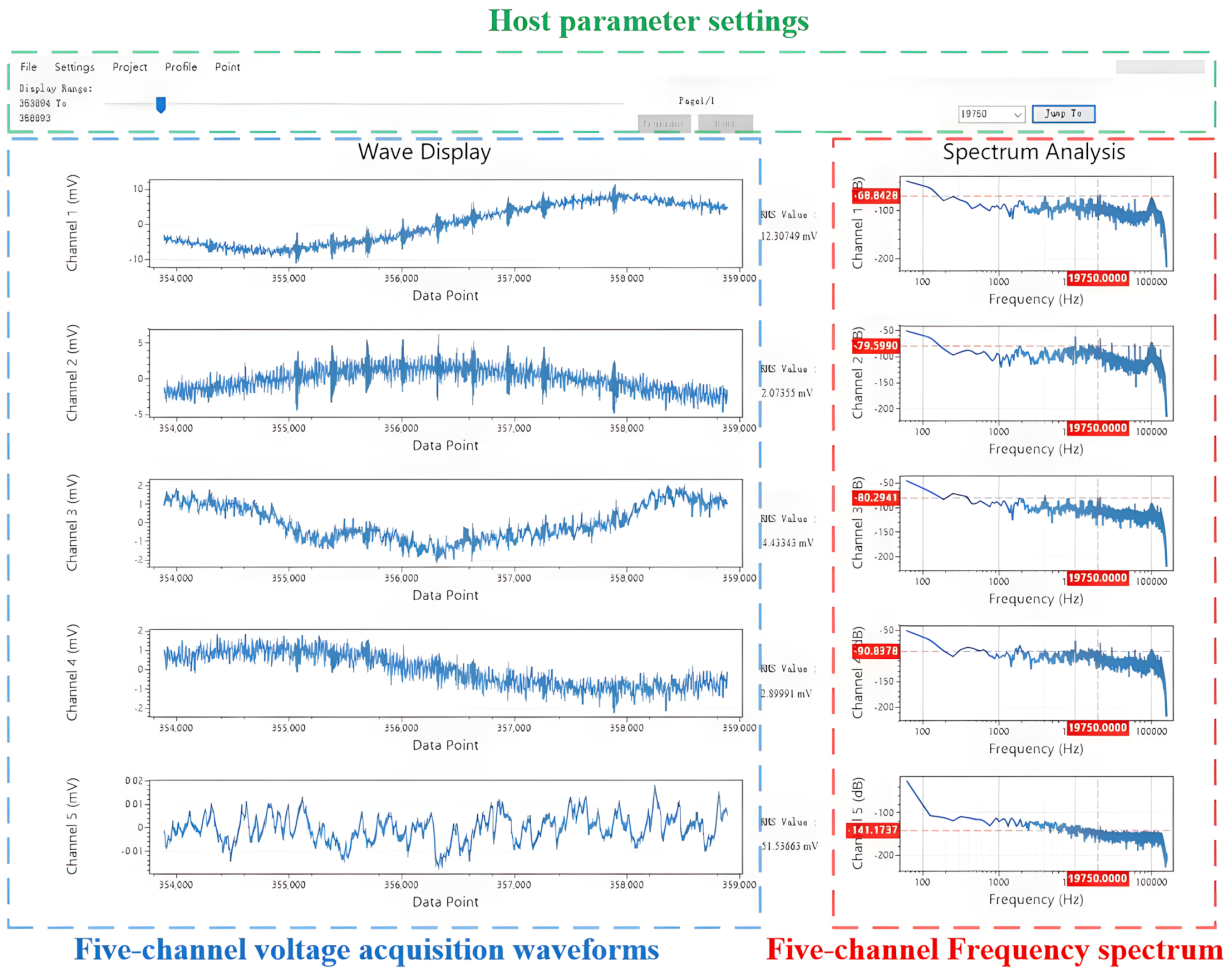
Network communication process diagram.

Furthermore, to test the system’s capability for capturing weak, long-distance signals, a Very Low-Frequency (VLF) reception test was conducted. As a representative result, the UAEM system successfully received electromagnetic signals transmitted from the Harold E. Holt VLF station in Australia. This station, jointly operated by the United States Navy and the Royal Australian Navy, provides communication services to surface vessels and submarines in the Western Pacific and Eastern Indian Oceans. The transmitted signal has a nominal frequency of 19.8 kHz. A screenshot from the host computer’s software interface, displaying the reception of this signal, is shown in Figure 12. The system’s host software is capable of simultaneously displaying multi-channel time-domain waveforms and their corresponding frequency-domain spectra. By configuring parameters such as the frequency display range and the number of FFT points, a user can monitor a real-time graphical representation of the data currently being acquired.

### 4.3. Comparative Analysis of Geophysical Prospecting Results

**Figure 13:**
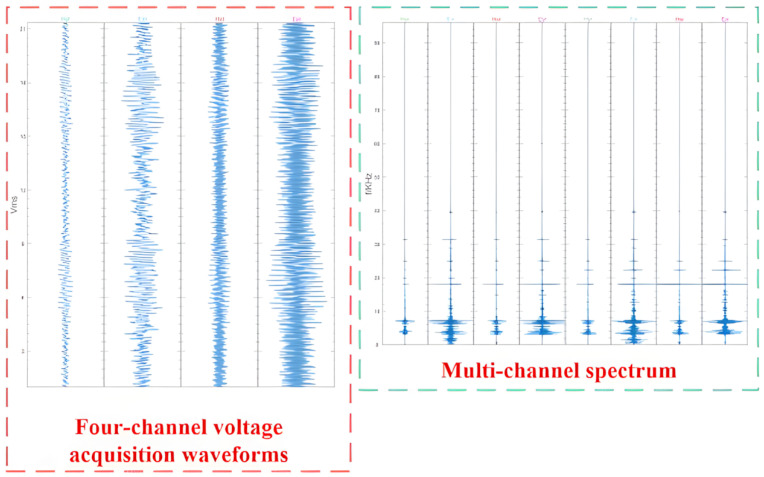
Upper-computer waveform display interface design.

To provide a direct performance benchmark against conventional ground electromagnetic (EM) instrumentation, the Geometrics Stratagem EH4 system, a well-regarded commercial instrument, was deployed concurrently during our field tests. Comparative analysis reveals that the EH4 (Geometrics, Inc., San Jose, CA, USA)system operates within a frequency range of 10 Hz to 100 kHz. While this band is sufficient for many conventional EM surveys, it presents clear limitations in high-resolution, near-surface investigations. In contrast, our UAEM system features an extended frequency range from 1 Hz to 1000 kHz. This not only encompasses the entire operational band of the EH4 but also significantly enhances the receiver’s response to high-frequency signals, which is critical for the detailed characterization of shallow geological targets. The performance disparity is also evident in the real-time data visualization. As shown in Figure 12, the UAEM receiver provided richer spectral information and a more intuitive display of signal strength distribution. The data presented by the EH4 system (Figure 13), conversely, was comparatively sparse, making it less conducive to rapid on-site analysis and quality control. Furthermore, a critical operational distinction is the EH4 system’s reliance on a dedicated, large-scale artificial transmitter. The logistics associated with its transport, deployment, and maintenance lead to higher survey costs and lower field efficiency. Our UAEM system circumvents this requirement by leveraging pre-existing anthropogenic field sources, such as broadcast radio and VLF naval stations, which dramatically streamlines the operational workflow.

## 5. Conclusions

This paper presents a vehicle-mounted Ultra-Audio frequency Electromagnetic (UAEM) survey system designed for rapid, electrode-free “stop-and-go” profiling. Operating in a Signals of Opportunity (SOOP) mode—utilizing pre-existing background field sources such as AM broadcasts and VLF signals—this approach obviates the need for the bulky, dedicated transmitters required by conventional Controlled-Source Radio Magnetotellurics (CSRMT), thereby dramatically streamlining field logistics and reducing operational costs. Comprehensive laboratory tests demonstrate that the developed receiver console features an ultra-wide analog-to-digital acquisition bandwidth (1 Hz–1000 kHz) and a high dynamic range of 143 dB. In actual field operations, operators connect specifically matched electromagnetic sensors to the console depending on the targeted frequency band. Furthermore, data acquisition is conducted strictly while the vehicle is temporarily stationary, fundamentally circumventing the insurmountable physical problem of motion-induced magnetic noise. During a field survey over the Beijing Metro Changping Line tunnel, the system successfully imaged the known subsurface target, validating the effectiveness of this hardware setup for high-resolution surveys in complex urban environments.

Future research will focus on two key directions. First is the continued optimization of the hardware to enhance system integration and portability, enabling efficient deployment in more challenging terrains. Second is the expansion of its application to new domains, with a particular focus on investigating its potential in areas such as marine electromagnetic exploration.

## Figures and Tables

**Table 1:** UAEM electromagnetic measurement system frequency range test results.

Frequency (Hz)	Voltage Amplitude (V)	Attenuation Factor (dB)
1	1.00076	0.0066
10	1.00044	0.0038
100	1.00078	0.0068
1000	0.99885	−0.010
10,000	0.999467	−0.046
100,000	0.99617	−0.033
600,000	0.96660	−0.30
1,000,000	0.95021	−0.4410

## Data Availability

The original contributions presented in this study are included in the article. Further inquiries can be directed to the corresponding author.
